# Exploring practices to enhance benefits and reduce risks of chemsex among gay, bisexual, and other men who have sex with men: A meta-ethnography

**DOI:** 10.1016/j.drugpo.2024.104398

**Published:** 2024-03-30

**Authors:** Drew E. Hawkinson, T. Charles Witzel, Mitzy Gafos

**Affiliations:** a London School of Hygiene and Tropical Medicine, 15-17 Tavistock Place, London, WC1H 9SH, United Kingdom; b Institute for Global Health, University College London, Gower Street, London, WC1E 6BT, United Kingdom; c Center of Excellence in Research on Gender, Sexuality and Health, Mahidol University, 999 Phutthamonthon Sai 4 Rd, Nakhon Pathom, Thailand; d Department of Global Health and Development, London School of Hygiene and Tropical Medicine, 15-17 Tavistock Place, London, WC1H 9SH, United Kingdom

**Keywords:** Gay, bisexual, and other men who have sex with men, Chemsex, Harm reduction, Meta-ethnography

## Abstract

**Background:**

Chemsex is the intentional combining of specific drugs with sex, primarily by gay, bisexual, and other men who have sex with men (GBMSM), to enhance intimacy, pleasure, and prolong sexual sessions. Practices vary across geographic and social settings. Participants report benefits and risks of chemsex. Studies have previously reviewed chemsex practices and harm reduction interventions separately. This review aims to examine both together by describing and understanding practices that men employ to navigate the perceived benefits and risks of chemsex.

**Methods:**

We conducted a systematic meta-ethnographic review of published qualitative literature, screening titles, abstracts, and full texts on defined inclusion and exclusion criteria. Using reciprocal and refutational translation techniques, we analysed study participants’ (first-order) and researchers’ (second-order) accounts of benefit-enhancing and risk-reducing chemsex practices. Finally, we employed line-of-argument synthesis techniques to develop our own higher-level interpretations (third-order constructs) of these chemsex practices.

**Results:**

Our search yielded 6356 records, from which, we included 23 articles in our review. Most studies were conducted in high-income Western countries. Across studies, participants acted at the individual, interpersonal, and community levels to enhance benefits and reduce risks, which made up our third-order constructs. Eight themes emerged from first- and second-order constructs to describe these practices, which included personal preparation, personal boundaries, biomedical measures, structured use of drugs, leaning on partners, injecting practices, group organising, watching out for others, and teaching and learning. Contextual factors like trust, agency, access, stigma, and setting moderated whether and how participants engaged in these practices, and if practices enhanced benefits or reduced risks.

**Conclusion:**

Health promotion programmes and research focused on chemsex must account for the benefits and the risks that GBMSM associate with this type of sexualised drug use and target the moderating factors that shape the practices they employ to navigate these benefits and risks.

## Introduction

Chemsex, also referred to as “party-n-play” or “hi-fun,” is a specific type of sexualised drug use (SDU). While SDU encompasses a wide range of sexual activities among partners while under the influence of drugs, chemsex, a term that developed more recently, is a practice among gay, bisexual, and other men who have sex with men (GBMSM) in which specific drugs are intentionally combined with sex to facilitate, sustain, and intensify prolonged sexual experiences (Hibbert et al., 2021; Maxwell et al., 2019; Stuart, 2019; Witzel et al., 2023). Research on chemsex first emerged in Europe, North America, and Australia; however, recent studies conducted with GBMSM show evidence of chemsex in the Middle East, East and Southeast Asia, South America, and across Africa, primarily concentrated in large urban centres (Aidsmap, 2019; Cassim, 2019; Jalil et al., 2022; Nevendorff et al., 2023).

Chemsex drugs (“chems”) vary across geographic and social contexts, but typically include methamphetamine (crystal meth), mephedrone, and gamma-Hydroxybutyric acid (GHB/GBL). In some settings, powder and crack cocaine, ketamine, and 3,4-Methylenedioxymetham-phetamine (MDMA/ecstasy) are also used as chemsex drugs (Amundsen et al., 2023; Bourne, 2015; Maxwell et al., 2019; Tomkins et al., 2019). GBMSM may consume drugs through snorting, smoking, or anal insertion (Bourne et al., 2014; Maxwell et al., 2019). A subset of GBMSM inject chemsex drugs, a practice known as “slamming” (Glass et al., 2017; Scheibein et al., 2021).

GBMSM engage in chemsex in various settings. These include anonymous sessions typically organised online by “hosts” through social media and/or geosocial networking apps (apps), semi-closed parties among close social networks, more casual and unplanned chemsex (sometimes termed “chill-sex” or “chillouts”), and chemsex at sex-on-premise venues (SPVs) like saunas (Ahmed et al., 2016; Londoner, 2016; Pires et al., 2022; Santoro et al., 2020). Across these settings, sessions may include different and multiple sexual behaviours, such as anal sex, oral sex, group sex, fisting, and use of sex toys (Maxwell et al., 2019; Tomkins et al., 2019).

GBMSM associate many benefits with chemsex: enhanced sexual stamina, physical pleasure, and greater intimacy with partners (Flores-Aranda et al., 2019; Graf et al., 2018; Tan et al., 2021). While not specific to GBMSM, recent work in Australia and Greece explained motivations for SDU through Michel Foucault’s theory of technologies of the self, stating that drugs assist lesbian, gay, bisexual, transgender, and other queer (LGBTQ+) people in exploring and reclaiming their sexuality by facilitating optimal cognitive, social, and physical conditions for sex and intimacy (Pienaar et al., 2020; Poulios, 2022; Race et al., 2016). This framing aligns with other researchers’ interpretation of chemsex as a desired form of disinhibition for GBMSM in which they are liberated from conventions and stigmas of sex and sociality (Drysdale et al., 2020; Melendez-Torres & Bonell, 2017; Nimbi et al., 2020; Schroeder et al., 2022; Weatherburn et al., 2017). In doing so, researchers describe that men develop a sense of community connectedness, another benefit of chemsex, especially more marginalised GBMSM like men living with HIV or men who inject drugs (Dennermalm et al., 2021; Flores-Aranda et al., 2019; Leyva-Moral et al., 2023; Milhet et al., 2019; Nimbi et al., 2020; Santoro et al., 2020; Weatherburn et al., 2017). In Southeast Asia, researchers have found that GBMSM also associate the chemsex community with high social status, seeing it as “Western”, “wealthy”, and “glamourous” (Tan et al., 2018; Witzel et al., 2023).

Evidence on the public health implications of chemsex is mixed. Researchers have reported that chemsex is associated with condomless anal intercourse (CAI) and therefore may be a risk factor for transmission of HIV, bloodborne viruses (BBVs), and other sexually transmitted infections (STIs) (Edmundson et al., 2018; Glynn et al., 2018; Melendez-Torres & Bourne, 2016; Pufall et al., 2018). However, other systematic reviews have demonstrated that the majority of evidence linking chemsex with HIV and STI transmission is purely associative, and thus may be influenced by sampling bias and unobserved confounders (Maxwell et al., 2019; Tomkins et al., 2019). While study participants have acknowledged that HIV, BBV, and STI transmission are potential consequences of chemsex, evidence varies in terms of whether they perceive these infections as substantial risks given that, with adequate healthcare access, they can be easily preventable, treatable, and/or curable (Bourne et al., 2015b; Drysdale et al., 2021; Herrijgers et al., 2020). Studies have also reported associations between chemsex and poor mental health, though researchers have questioned the strength and direction of these associations (Berg et al., 2020; Bohn et al., 2020; Bourne et al., 2014; Halkitis & Singer, 2018; Peyriere et al., 2023). Further evidence suggests some GBMSM who engage in chemsex are at risk of drug dependency, drug-induced psychosis, and fatal overdose—especially GBMSM who use GHB and who inject drugs (Dijkstra et al., 2021; Drevin et al., 2021; Evers et al., 2020; Joyce et al., 2018; Moreno-Gámez et al., 2022; Witzel et al., 2023). Finally, there is emerging evidence detailing risk of sexual violence and rape in chemsex settings (Druckler et al., 2021; Javaid, 2018; Wilkerson et al., 2021).

Previous studies have examined and advocated for harm reduction efforts targeting GBMSM who engage in chemsex, yet no work has reviewed and synthesised studies of GBMSM’s own practices for navigating the benefits and risks of chemsex across diverse geographic and social contexts (Bourne et al., 2015a; Platteau et al., 2023; Stardust et al., 2018; Strong et al., 2022; Tan et al., 2018). Furthermore, ideas of harm reduction for chemsex may fail to consider GBMSM’s motivations for chemsex and how underlying risk environments at the individual, interpersonal, community, and societal levels serve as barriers to these efforts (Rhodes, 2002; Tan et al., 2018; Witzel et al., 2023).

This review aims to explore, using meta-ethnography, how and why men engage in specific practices to enhance potential benefits and reduce potential risks of chemsex. While one previous meta-ethnography of chemsex posits that sessions are “littoral spaces” where substance use facilitates the pushing of social, sensory, and sexual limits to bring about potential benefits and harms, it did not aim to explain the underlying practices men employ to navigate these benefits and harms (Melendez-Torres & Bonell, 2017). In conceptualising how GBMSM’s practices are shaped by their individual, geographic, and social contexts, our review can help inform harm reduction efforts for chemsex and reframe the public health and research agenda to be more grounded in GBMSM’s diverse, multifaceted, and even seemingly paradoxical, chemsex practices.

## Methods

### Design

This review is registered with PROSPERO (ID: CRD42023479011) and reported in line with recently developed eMERGe guidelines for meta-ethnography (France et al., 2019). Meta-ethnography, developed by George W. Noblit and R. Dwight Hare, is a qualitative synthesis methodology through which researchers translate accounts of phenomena across different studies, charting how interpretations of study participants (first-order constructs) lead to interpretations of study authors (second-order constructs), and finally, lead to higher-level interpretations of phenomena through the reviewers’ own synthesis (third-order constructs) (Noblit & Hare, 1988). We chose meta-ethnography because it allowed us to systematically analyse the complexities of chemsex practices as described by GBMSM and researchers, separately, helping us develop a deeper conceptual understanding of how GBMSM navigate chemsex benefits and harms through their practices.

### Ethics approval

No ethics approval was sought for this review, as it did not involve human or animal participants.

### Eligibility criteria

We included studies that: sampled GBMSM with direct experience engaging in chemsex in any region of the world; described practices GBMSM employed to enhance the benefits and/or reduce the risks of chemsex; reported qualitative data from one-on-one interviews, group interviews, focus groups, and/or ethnography; and were published in peer-reviewed academic journals in English or with an available English translation. We defined chemsex as the intentional consumption (including injection) of one or more of the following substances by GBMSM while engaging in sexual behaviour to prolong, sustain, and intensify their sexual experience: amphetamines, methamphetamines, GHB/GBL, powder and crack cocaine, ketamine, MDMA/ecstasy, synthetic cathinones (including mephedrone), and other new psychoactive substances (NPS). We excluded caffeine, alcohol, tobacco, and cannabis from our definition of chemsex drugs, as use of these drugs when not combined with other chemsex drugs is systematically excluded in researchers’ definitions of chemsex (Amundsen et al., 2023). Additionally, we excluded sildenafil and amyl/alkyl nitrates. While these drugs have been grouped under the chemsex umbrella in some settings, other research shows that many GBMSM view them as separate from chemsex drugs, because their purpose is primarily to assist in bodily functions associated with sexual intercourse and rather than to facilitate prolonged altered psychoactive states (Amundsen et al., 2023; Evers et al., 2019; Hammoud et al., 2018; Schwartz et al., 2020; Yu & Lasco, 2023).

During full text screening, we excluded studies that reported data from participants who were not GBMSM alongside data from GBMSM, as interpretations were not able to be attributed solely to the population of interest. We also excluded studies where the sample was known to include GBMSM who engage in sex work, as the potential benefits and risks of their engagement in chemsex may be influenced by the specific context of sex work; however, some included study samples may have contained GBMSM who engage in sex work.

### Search

Using the SPIDER tool (**s**ample population, **p**henomenon of **i**nterest, study **d**esign, **e**valuation, **r**esearch type) for systematic reviews we developed four components for our overall search strategy: GBMSM, sexual intercourse, drug use, and qualitative research. We performed searches in CINAHL Complete, EMBASE, MEDLINE, PsycINFO, and Web of Science, limiting results to studies published after 1 January 2010, as previous reviews of chemsex reasoned that chemsex practices and contexts changed dramatically after the introduction of apps in 2009 (Amundsen et al., 2023; Tomkins et al., 2019). Our full MEDLINE search strategy is included as supplementary material.

### Study selection

Using EPPI-Reviewer, DEH screened all deduplicated titles and abstracts against eligibility criteria (Thomas et al., 2010). TCW independently screened a sample of 10% of excluded citations to ensure inter-rater consistency. Next, DEH screened full texts of all remaining citations and TCW independently checked 10% of full texts screened out and in for consistency. All eligible articles were citation searched backwards and forwards and new citations from this search were screened against eligibility criteria, resulting in the final articles included in this review.

### Quality assessment

We used the Critical Appraisal Skills Programme (CASP) tool for qualitative studies to appraise the quality of each study, assessing the aims, methodology, data collection, data analysis, ethical considerations, reflexivity, reports of findings, and value of each study in relation to the aim of this review (Atkins et al., 2008; CASP; Sattar et al., 2021). Additionally, we assessed if studies discussed chemsex in a non-judgmental way, since reporting of chemsex has historically been subject to negative bias in academic literature and in public media (Hakim, 2019).

### Data extraction and synthesis

Using NVivo 12, DEH extracted all first-order data (things said by study participants) and second-order data (things said by researchers) from the studies, coding them under four parent nodes: 1) first-order data on practices to enhance benefits of chemsex; 2) first-order data on practices to reduce risks of chemsex; 3) second-order data on practices to enhance benefits of chemsex; 4) second-order data on practices to reduce risks of chemsex (Britten et al., 2002; France et al., 2019). Next, we analysed our data inductively using reciprocal and refutational translation techniques to trace consistencies and contradictions across studies’ accounts, as well as across orders of data, and create first- and second-order constructs. We developed third-order constructs by using line-of-argument techniques to synthesise these translations and conceptualise how GBMSM organise their benefit-enhancing and risk-reducing practices at different levels of action. We structured our third-order constructs using the first three levels of the Social Ecological Model (SEM): individual level, interpersonal level, and community level (Bronfenbrenner, 1981). This also allowed us to explore alternative explanations of practices at each of these levels of action and examine how individual, geographic, and social contexts impacted each study’s thematic contributions (Sattar et al., 2021).

### Reflexivity

Several of the review authors have backgrounds as harm reduction researchers and practitioners, thus, we were specifically interested in how GBMSM themselves understand and navigate benefits and risks of chemsex. We analysed data from a more interpretivist lens. In practice, this meant we interpreted chemsex practices as enhancing or reducing benefits and risks based on their meanings to study participants and researchers, which may be counter to medical/scientific opinion. Furthermore, given our aim, we analysed practices explored in this meta-ethnography through the lens of benefit and risk navigation. Therefore, our study may limit interpretations of practices outside of this lens.

## Findings

### Study selection

**Fig. 1:**
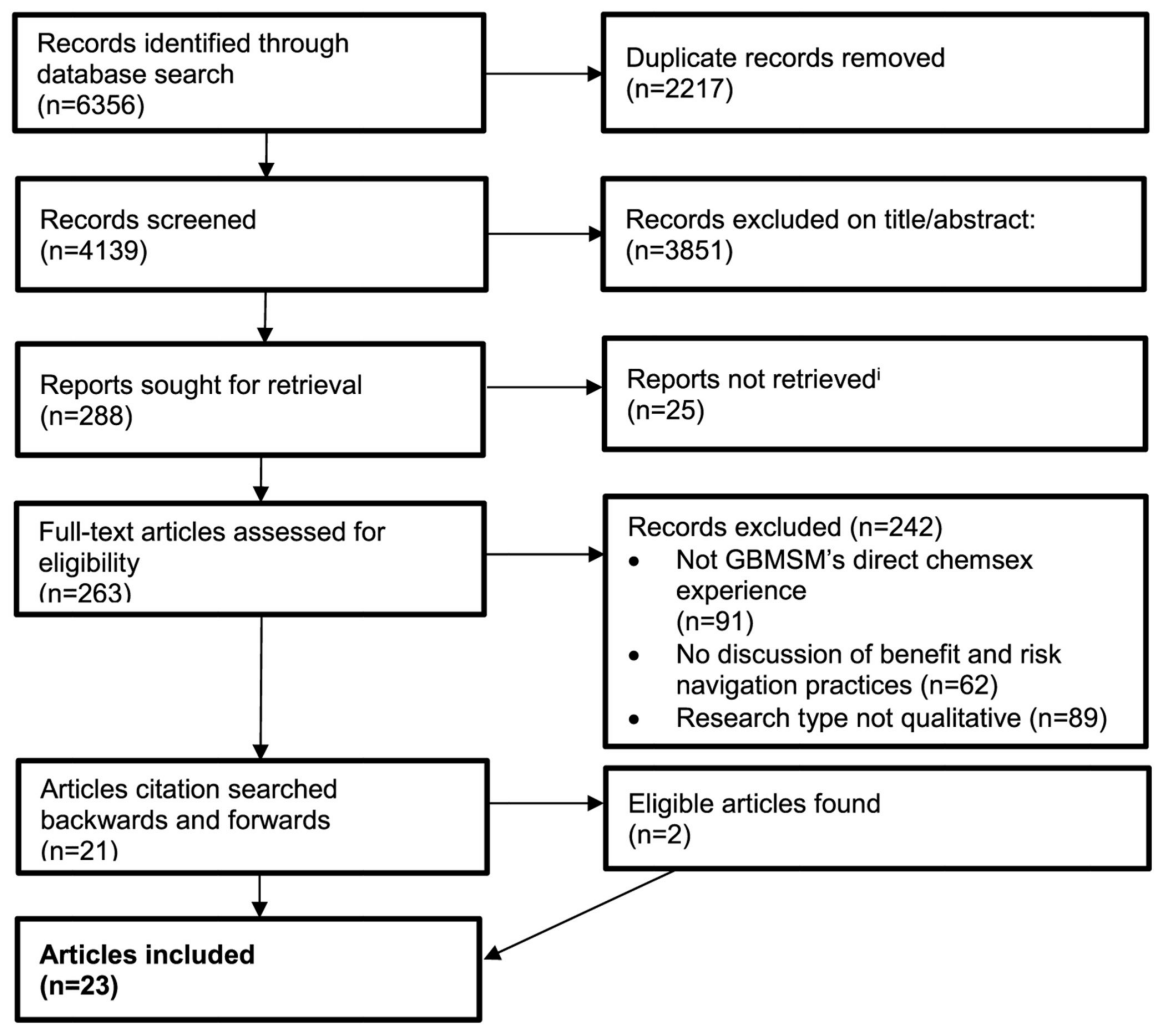
PRISMA diagram. ^i^Reports were not able to be retrieved for various reasons, including reports not being able to be accessed through the institutional library’s local collection or via interlibrary loan, lack of response from authors or contacts, and broken report links.

The search resulted in 6356 records (4139 unique records after deduplication); 263 reports remained for full text screening, of which 21 were eligible. Two additional articles were added after backward and forward citation searching (Fig. 1).

### Study characteristics

The 23 articles in this review came from eighteen unique studies and represent data from 513 participants, overall. Most articles came from studies conducted in Europe (13), followed by North America (4), Australia (4), and Southeast Asia (2). One article was originally published in Spanish and was translated into English (Leyva-Moral et al., 2023). All other articles were originally published in English. Studies varied in their inclusion of different drugs used by the sample population, with some only including methamphetamine; some only meth-amphetamine, mephedrone and GHB/GBL; and some including other drugs like ketamine, cocaine, and MDMA/ecstasy. Three studies only included GBMSM who injected drugs, examining this as a specific chemsex practice (Amaro, 2016; Race et al., 2021; Schroeder et al., 2022).

The studies had various aims and objectives, ranging from understanding forms of pleasure derived from chemsex to exploring harm reduction strategies among participants. Two studies specifically investigated the use of PrEP as an HIV prevention strategy, discussing participants’ perceptions and practices related to PrEP in the context of chemsex (Closson et al., 2018; Maxwell et al., 2022).

### Quality of studies

All studies were of acceptable quality. Only four of the 23 articles included statements of reflexivity and the relationship of the researchers to the sample population (Amaro, 2016; Dennermalm et al., 2021; Guadamuz & Boonmongkon, 2018; Santoro et al., 2020). Four articles did not report any ethical considerations or ethics board approval (Drysdale et al., 2020; Leyva-Moral et al., 2023; Milhet et al., 2019; Santoro et al., 2020). Three articles did not describe the participant recruitment strategies employed (Herrijgers et al., 2020; Milhet et al., 2019; Nimbi et al., 2022). One article did not include a description of the data analysis process (Amaro, 2016). One did not have a clear statement of findings (Milhet et al., 2019). Finally, three articles described chemsex only as harmful without acknowledging potential benefits GBMSM may derive from it (Herrijgers et al., 2020; Nimbi et al., 2020; Van Hout et al., 2019). Table 1 shows the full characteristics of each study, in order of rank for overall quality.

### Constructs

While we analysed data using an inductive approach, we organised the themes we generated from our translations of participants’ and researchers’ accounts of chemsex practices at the individual-, interpersonal-, and community-level of action. Our third-order constructs are reported below according to these levels. Within each third-order construct, we present supporting evidence from first- and second-order constructs, specifying how participants’ accounts (first-order) of practices are interpreted by researchers in their accounts (second-order) of practices. No studies detailed any practices that were actioned by GBMSM at the societal level; therefore, we did not include this level as a third-order construct. In explaining our synthesis of third-order constructs, we identify the factors that moderate GBMSM’s practices across all three levels of action. Table 2 presents third-order constructs with a summary of linked first- and second-order constructs.

### Construct 1: individual control

At the individual level, participants attempted to control their chemsex experience. Individual-level action was seen by men to reduce their exposure to perceived chemsex risks, such as HIV acquisition and drug dependency, by asserting agency to control the type of chemsex they had. Asserting this agency also allowed men to control the desired benefits of chemsex, such sexual satisfaction and disinhibition. However, practices that participants employed to control their chemsex experiences were moderated by participants’ ability to assert this agency over themselves or others and their access to specific health resources. These practices included planning their engagement in chemsex sessions, creating personal boundaries, utilising biomedical tools (PrEP, clean injecting equipment), and structuring their use of drugs.

Across studies, participants engaged in preparatory activities before chemsex, such as buying food and drinks, measuring and preparing drug doses, and procuring clean injecting equipment, to avert immediate risks such as dehydration, exhaustion, overdose, and potential transmission of BBVs during chemsex (Bourne et al., 2015b; Drysdale et al., 2021; Herrijgers et al., 2020; Leyva-Moral et al., 2023; Santoro et al., 2020; Souleymanov et al., 2021):

*“I don’t mix substances, I drink [water] a lot when I take drugs and I always prepare my doses on my own.”* [participant (Nimbi et al., 2020) p.1877]

Some participants strategically scheduled sessions over the weekend to have time to rest and manage comedown symptoms. Others deliberately planned “exit strategies,” scheduling activities like work, exercise, or social events following chemsex sessions (Amaro, 2016; Bourne et al., 2015a; Dennermalm et al., 2021; Guadamuz & Boonmongkon, 2018; Herrijgers et al., 2020). Participants also used planning to limit the frequency with which they engaged in chemsex:

*“I would always take a break afterwards and let myself come down… And, therefore, the next time I used a couple of weeks later, I’ve already come up and bounced back, so the next rush would be just as it was the first time.”* [participant (Drysdale et al., 2021) p.4]

As described, this strategy also enhanced benefits of chemsex, allowing participants to continue to feel the desired “rush” of euphoria (Amaro, 2016; Drysdale et al., 2021; Flores-Aranda et al., 2019; Nimbi et al., 2022).

Participants also contained their experience through personal boundaries of their drug use and chemsex. For some, this meant not injecting drugs, never procuring drugs themselves to prevent use outside of chemsex sessions, never sharing sex toys or injecting equipment, and/ or maintaining a level of lucidity during chemsex. These actions eased participants’ anxieties about potential risks—STI, HIV, and BBV transmission; overdose; drug dependency; sexual assault—which researchers interpreted as helping them enjoy chemsex more because they felt safe in being disinhibited (Herrijgers et al., 2020; Leyva-Moral et al., 2023; Nimbi et al., 2022; Schroeder et al., 2022). However, through their analyses, researchers found that it became more difficult for participants to set and adhere to these boundaries in contexts where power imbalances existed between participants, such as in anonymous sessions where hosts supply participants with drugs or when hosts have greater socioeconomic status than other participants (Drysdale et al., 2020; Guadamuz & Boonmongkon, 2018; Lim et al., 2018; Santoro et al., 2020).

*“…some participants described feeling indebted to other people in PnP [Party ‘n Play] settings. Lacking independence led to experiences of constrained agency in setting the tone, which at times felt threatening to them.”* [researcher (Schroeder et al., 2022) p.5]

GBMSM’s “constrained agency” over their actions due to the norms of the setting in which they practice chemsex serves as a barrier to self-control, moderating men’s ability to engage in individual-level practices like boundary setting.

Participants also used biomedical tools to reduce their risk of STIs, HIV, and other BBVs. One man in an Australian study spoke of taking antibiotics for chlamydia and gonorrhoea as post-exposure prophylaxis after chemsex sessions, a method known as “DoxyPEP” (Drysdale et al., 2021). In studies set in areas with greater access to PrEP and HIV medication (ART), participants described use of PrEP or adherence to ART as important individual responsibilities of each chemsex participant (Dennermalm et al., 2021; Drysdale et al., 2021; Flores-Aranda et al., 2019; Herrijgers et al., 2020; Maxwell et al., 2022; Souleymanov et al., 2019).

*“I’m HIV negative and I use PrEP and that’s enough for me… it doesn’t matter what else is happening outside of me, because I know I’ve got me.”* [participant (Drysdale et al., 2021) p.3]

In these contexts, participants did not perceive STIs or HIV as serious risks because they were easily preventable, treatable, and/or curable (Bourne et al., 2015b; Closson et al., 2018; Guadamuz & Boonmongkon, 2018; Lim et al., 2018; Maxwell et al., 2019; Nimbi et al., 2020). This was not true of GBMSM in areas with less access to these biomedical tools and greater stigma of HIV and STIs, such as in Southeast Asia and Sweden (Dennermalm et al., 2021; Guadamuz & Boonmongkon P, 2018; Lim et al., 2018).

Participants who used PrEP or were on ART also felt these tools enhanced the benefits of chemsex because they helped participants feel more confident in having CAI, which participants said enhanced physical pleasure and intimacy:

*“I had these things [CAI] that I wanted to try but I was always aware that I didn’t want to catch HIV. PrEP just seemed to be the key in allowing me to experience my*–*well my fantasies really.”* [participant (Maxwell et al., 2022) p.3]

In this way, GBMSM’s use of biomedical tools like PrEP and ART as an individual-level practice was moderated by access to these tools. Furthermore, access to these tools moderated if men perceived this practice as enhancing the benefits of chemsex, by giving men the confidence to have CAI and feel increased intimacy and pleasure, and/or reducing the risks of chemsex by preventing transmission of HIV.

Participants spoke about structuring their use of drugs as an important strategy to avoid the more acutely felt risks of drug dependency and overdose, which researchers then interpreted as a form of self-control. This included measuring and keeping a log of drug dosing, which was especially important for participants using GHB/GBL because of the fine line between achieving desired effects of the drug and overdose (Ahmed et al., 2016; Dennermalm et al., 2021; Drysdale et al., 2021; Herrijgers et al., 2020; Van Hout et al., 2019).

Participants across studies had contrasting views on whether injecting drugs was risk-reducing or risk-enhancing, based primarily on their own histories. Participants without a history of injecting drugs often stated that they did not inject drugs because they perceived certain risks, like drug dependency and overdose, as greater if they engaged in this practice (Ahmed et al., 2016; Amaro, 2016; Drysdale K et al., 2021). Ahmed et al. interpreted this framing as a by-product of internalised stigma attached to people who inject heroin (2016). This internal stigma moderated men’s choice on how to consume drugs and whether they viewed certain individual-level drug consumption practices as reducing or enhancing risks. Conversely, participants who injected drugs as part of their chemsex practice often spoke about their choice to inject drugs as a means of controlling their use and reducing those very same risks while also intensifying arousal and belonging, as described by Race et al.:

*“They report that injecting crystal rather than smoking it enables them not only to experience something extraordinary*—*an exhilarating sense of erotic connection in a troubled world*—*but (more pragmatically), to draw a temporally bounded circle around the consumption event*—*something they would find harder to accomplish using other methods.”* [researcher (Race et al., 2021) p.16]

As interpreted by researchers (second-order), because of the immediate effect, injecting chemsex drugs was seen as a strategy to confine drug use spatially, mentally, and temporally to chemsex settings. When internal stigma around injection drug use was less present due to their history of injecting, men saw injecting drugs as both reducing risks and enhancing benefits of chemsex.

### Construct 2: interpersonal relationships

Participants also negotiated practices at the interpersonal level. Often, both participants and researchers viewed practices originating from these interpersonal relationships as a way to achieve enhanced intimacy during chemsex and allow participants to feel safe experiencing satisfying disinhibition. At the same time, many participants used these interpersonal relationships as tools to support themselves in reducing various risks of chemsex, like drug dependency and HIV/BBV acquisition. Trust in partners and agency to act, both as individuals within the relationship and as a pair, moderated whether participants engaged in practices to enhance benefits and reduce risks of chemsex. Furthermore, without trust and agency, the same practices had the opposite effects—reducing benefits and enhancing risks. Two specific relationships were important in this construct: that between romantic partners and that between injectors and those they inject with drugs.

Participants who engaged in chemsex with their romantic partners, either one-on-one or in group settings, negotiated certain practices together so they were safe from risks like overdose and dependency (Drysdale et al., 2020, 2021). This included one partner staying more lucid to watch out for the other and partners helping each other to limit engagement in chemsex to make sure it did not interfere with other aspects of their lives (Drysdale et al., 2020, 2021; Race et al., 2021). Partners also supported each other in taking PrEP if they were engaging in group sex:

*“…they reminded each other to take their [PrEP] doses before and after chemsex sessions when they involved casual sex partners.”* [researcher (Maxwell et al., 2022) p.4]

These risk-reducing, interpersonal-level practices, however, were moderated by GBMSM’s agency within relationships and trust in their partners. As Amaro’s study of relationships in the context of chemsex shows, some participants felt that when they lost agency in their relationship, partners pushed them past their own personal boundaries and enhanced risk of dependency (Amaro, 2016). In his second-order account, Amaro interprets how relationships offer opportunities for both potential outcomes (reduction of risk and enhancement of risk):

*“… symbiotic relationships can indeed become vectors of risk-taking. But romantic relationships can also provide crucial symbolic and material support to navigate drug use in a way that reduces harm.”* [researcher (Amaro, 2016) p.220]

This complexity was also seen in participants’ relationships with men who inject them with drugs during chemsex sessions. Many participants found being injected by another person as an erotic practice that enhanced attraction and intimacy:

*“…say we both […] inject crystal as well*–*we have a blast together, that connection, it just makes you feel closer and it pushes the world away further, so your bubble is kind of like stronger.”* [participant (Race et al., 2021) p.8]

Participants who did not feel confident in injecting on their own also described that having someone else inject them was a practical way to reduce potential risks of poor injection (e.g. injuries, infections) (Bourne et al., 2015a; Drysdale et al., 2021; Nimbi et al., 2022). Not learning how to inject oneself was also a personal boundary that some participants created to avoid the risk of problematic drug use:

*“Sterling [pseudonym] discussed refraining from learning how to inject properly as a way of moderating his own drug use. Relying on others to inject him meant he was not tempted to do it by himself when he had drugs in the house.”* [researcher (Race et al., 2021) p.10]

In this way, people who inject others have unique risk reduction responsibilities when chemsex drugs are injected. In their second-order accounts, researchers reflected that this could increase potential risks of chemsex, such as overdose and transmission of HIV/BBVs because individuals may have less control over how they are being injected (Drysdale et al., 2021; Guadamuz & Boonmongkon, 2018; Race et al., 2021; Schroeder et al., 2022). Interestingly, in their first-order accounts, participants who injected drugs as part of their chemsex practice noted that they and those they inject with always use clean, unshared injecting equipment, though as researchers noted, understanding of and access to this injecting equipment varied (Bourne et al., 2015a; Drysdale et al., 2021; Guadamuz & Boonmongkon, 2018; Herrijgers et al., 2020; Souleymanov et al., 2021). Participants who learned how to inject themselves described doing so because of previous experience with bodily injury after being injected by others:

*“Some men reported that they had decided to learn how to inject to take responsibility for their own health but, more practically, to avoid being injured by others with poor injecting skills.”* [researcher (Drysdale et al., 2021) p.5]

Learning how to inject oneself versus being injected by others was a negotiated choice participants made moderated by trust in oneself as well as trust in others in relation to injecting. Further moderated by trust, this surrendering of control carried the potential to enhance or reduce the benefits, as well as enhance or reduce the risks, of chemsex.

### Construct 3: community care

Participants also acted at the community level, in and outside of group chemsex sessions, to look after one another. Men engaged in community-level practices together to reduce their and others’ risk of overdose, drug dependency, and sexual exploitation. Acting at the community level also enhanced participants’ sexual satisfaction and community connectedness. However, engagement in specific community care practices—group organising of chemsex sessions, monitoring others during sessions, and teaching others safer chemsex practices—was moderated by participants’ trust in others, agency to act in these communities, stigma from institutions, and the social and political norms of the settings where they engage in chemsex.

Hosts organise sessions through apps around specific aspects, such as HIV status of attendees (for serosorting purposes), types of drugs being used, and route of administration, to reduce potential risks of HIV transmission and overdose (Ahmed et al., 2016; Dennermalm et al., 2021; Drysdale et al., 2020, 2021; Santoro et al., 2020; Schroeder et al., 2022; Van Hout et al., 2019). When sessions were planned in advance, participants discussed and set boundaries with others before taking drugs (Drysdale et al., 2020; Santoro et al., 2020). Researchers explained that these supportive group practices were more common in semi-closed chemsex sessions among friends:

*“These are the kind of sessions where more deliberate strategies of protection (such as having pauses to eat and rest, arranging a time to end the party, or agreeing on how to take care of people who “pass out”) are put into practice. They are also the type of session where the serostatus of participants is more openly discussed.”* [researcher (Santoro et al., 2020) p.4]

In their second-order accounts, researchers interpreted these practices as norms of community care specific to chemsex settings where participants knew and trusted others. Trust and setting moderated these community-level practices. Participants monitored each other for overdose, limited others’ drug use if it appeared problematic, reminded each other to take PrEP or ART, and ensured others were taking care of their physical health by consuming electrolytes and water (Drysdale et al., 2021; Herrijgers et al., 2020; Nimbi et al., 2020; Santoro et al., 2020; Souleymanov et al., 2021; Van Hout et al., 2019).

*“My group of people that I usually play with, I sort of know their patterns… My friends are my community. I take away people’s keys. I have enough water on hand. I send email blasts letting them know that they have to take their medications.”* [participant (Souleymanov et al., 2021) p.3238]

However, in anonymous chemsex sessions where there was not pre-established trust between participants, researchers described how community norms shifted care responsibilities onto session hosts (Drysdale et al., 2020; Guadamuz & Boonmongkon, 2018; Herrijgers et al., 2020; Nimbi et al., 2022).

*“…[the host is] the person responsible when something goes wrong (*e.g., *in case of drug overdose). For this reason, the host limits [their own] alcohol and drug consumption to be able to monitor everyone.”* [researcher (Herrijgers et al., 2020) p.8]

This was true even in settings where hosts had greater social and financial capital than participants, like in Southeast Asia (Guadamuz & Boonmongkon, 2018; Lim et al., 2018). Therefore, this different setting where trust was not present among participants moderated community-level practices of group organising and monitoring.

From their analysis of participants’ first-order accounts, researchers found that fear of stigma and punishment from police and frontline healthcare professionals, something participants faced in several global settings, led men to rely on each other, rather than these institutions when problems arose, effectively serving to moderate community-level risk reduction practices (Guadamuz & Boonmongkon, 2018; Herrijgers et al., 2020; Leyva-Moral et al., 2023). Group organising and monitoring also helped foster trust among participants, which in turn moderated their ability to experience the disinhibitory benefits of chemsex and feel a sense of community connectedness (Dennermalm et al., 2021; Milhet et al., 2019). However, when trust among chemsex participants was lacking, participants felt that others were too focused on their own sexual satisfaction and would not engage in community care practices like monitoring others’ drug use or ensuring consent in sexual activities, moderating their ability to experience enhanced benefits and making them feel more at risk of overdose and sexual assault rather than reducing these risks (Bourne et al., 2015a; Dennermalm et al., 2021; Herrijgers et al., 2020; Lim et al., 2018).

Given the stigma and lack of access to appropriate services from mainstream institutions like substance use services, healthcare professionals, and law enforcement, participants also engaged in information sharing and teaching, as described by this man who learned skills from someone he met through chemsex:

*“He taught me how to do drugs… Even if you’ve read something on a drug website somewhere on how different drugs work, even then, you need someone to explain it to you.”* [participant (Dennermalm et al., 2021) p.8]

This community-level information sharing and teaching was seen in first- and second-order accounts as a strategy to reduce risk of overdose and dependency. In this way, stigma and access again moderated how men engaged in community-level teaching and learning. Researchers also interpreted teaching and learning as practice that enhanced participants’ sense of community connectedness (Amaro, 2016; Dennermalm et al., 2021; Herrijgers et al., 2020; Leyva-Moral et al., 2023; Nimbi et al., 2020; Souleymanov et al., 2021).

## Discussion

### Summary of findings

GBMSM associate various benefits and risks with chemsex. This meta-ethnography shows that men act at the individual, interpersonal, and community levels to enhance these benefits and reduce these risks. Certain contextual factors moderate men’s ability to engage in these practices. These moderators include men’s *trust* in partners and in themselves, *agency* to act in chemsex settings, *access* to health resources, *stigma* of chemsex practices and from institutions, and norms of the *settings* in which they practice chemsex. For example, in chemsex settings where individuals’ agency was diminished, such as by hosts with greater socioeconomic status or by coercive romantic partners, GBMSM were less able to engage in boundary-setting to dictate their own drug use.

These moderators also influence whether practices ultimately enhance benefits or reduce risks of chemsex. When participants trusted their partners, being injected by them enhanced benefits of chemsex (intimacy, arousal) and reduced potential harms (injury from poor injection technique); however, when participants did not trust their partners, this same practice enhanced risks and reduced benefits. Furthermore, engaging in various practices affected these moderators. As described, participants who used biomedical tools like PrEP felt increased agency to engage in CAI in chemsex settings because they knew they were protected from potential HIV infection and could better enjoy pleasure brought through CAI.

Our findings on the influential role that trust, agency, access, stigma, and setting play in moderating risk perception and risk reduction practices mirror research on harm reduction among people who use drugs, more broadly. For example, trust felt among partners who inject drugs together shapes individuals’ risk perceptions of sharing injecting equipment and being injected by their partners, moderating their choice of injecting practices (Morris et al., 2019). When examining risk environments in which drug use occurs, increased agency of people who use drugs at the microlevel (individual, interpersonal, and community levels) and macrolevel (societal level) not only moderates how effectively harm reduction is able to be practiced by people who use drugs, but also the types of harms and level of risk that are constructed within environments of drug use (Rhodes, 2009). Access to appropriate and supportive harm reduction materials plays a similar role in shaping and modifying the microlevel and macrolevel risk environments of drug use and blocking or supporting safer drug use practices. Throughout all contexts, stigma, at the individual-, interpersonal-, community-, and societal-levels, serves as a barrier for many to engage with harm reduction practices and services (e.g. drug checking) and strips protective factors of drug use (e.g. supportive community networks and availability of appropriate services), moderating the effectiveness of harm reduction practices (Davis et al., 2022; Treloar et al., 2021). Finally, harm reduction and health promotion research have long pointed to social norms as a key influencer of behaviour; settings in which behaviours, like drug use, occur determine the salience and accessibility of these norms, thus moderating these behaviours themselves (Edberg & Krieger, 2020).

### Implications

**Fig. 2:**
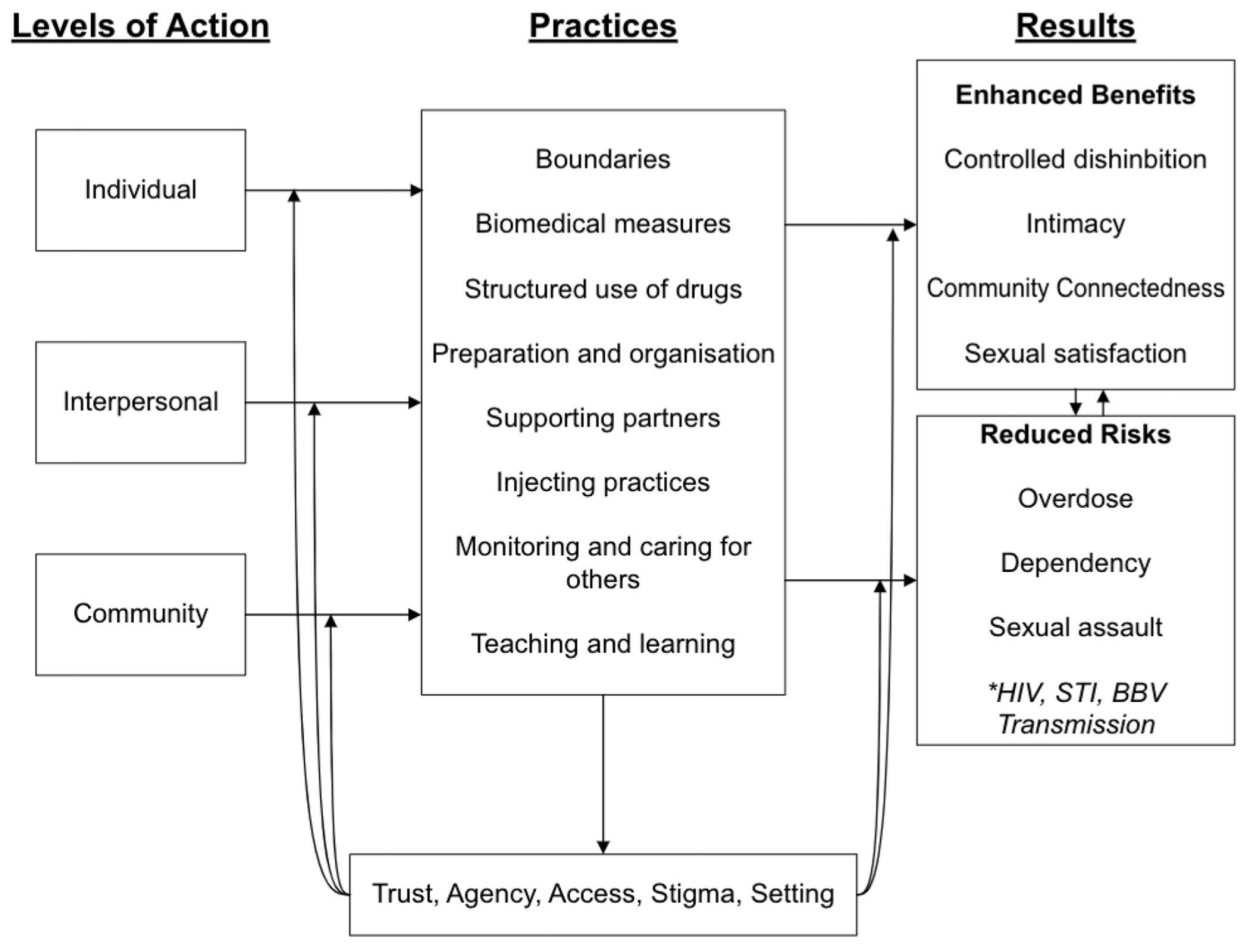
Conceptual model for how GBMSM navigate benefits and risks of chemsex. *Not all GBMSM view HIV, STI, and BBV transmission as a risk of chemsex, depending their own serostatus, use of biomedical prevention tools, access to testing and treatment, and felt stigma.

These findings corroborate previous studies detailing strategies employed by GBMSM to reduce risks of sex and chemsex (Dowsett et al., 2005; Gonçalves et al., 2016; Strong et al., 2022; Witzel et al., 2023). Furthermore, the moderating factors that influence GBMSM’s ability to engage in practices (trust, agency, access, stigma, setting) reflect those previously detailed in recent studies of chemsex harm reduction efforts (Tan et al., 2018; Witzel et al., 2023). Strong et al. recently reviewed harm reduction approaches to chemsex, categorising interventions by the type of harm they reduce (drug-related, STI-related, and HIV-related) (Strong et al., 2022). From our analysis, we propose a new conceptual model through which chemsex practices can be interpreted (Fig. 2). This model offers an initial illustration of how GBMSM navigate not only the risks, which are more extensive and less certain than described by Strong et al., but also the benefits of chemsex—a concept not thoroughly explored in previous literature. Researchers and public health practitioners may use this model to inform additional approaches to design and evaluate chemsex harm reduction efforts, building upon its concepts and tailoring them to their unique social and geographic contexts.

No previous studies have analysed chemsex practices using the SEM to organise practices by their levels of action. Tan et al. used the SEM to organise recommendations for interventions that may address various harms and needs of GBMSM who engage in chemsex in Singapore (Tan et al., 2018). While our findings reinforce those of Tan et al. in terms of the factors that influence men’s chemsex experience (stigma from institutions, access to harm reduction tools, agency between chemsex partners), our findings and model differ from and expand on Tan et al.’s in that they highlight the bottom-up practices already employed by GBMSM and the underlying mechanisms through which those practices both enhance benefits and reduce risks of chemsex (Tan et al., 2018).

In using the SEM as an organising tool, our model facilitates a deeper understanding of how chemsex practices are organised and actioned at various levels. GBMSM’s actions at each level (individual, interpersonal, and community) interact with benefit enhancement and risk reduction practices actioned at the other levels, as well. For example, enhancing community-level practices of group organising and monitoring may support individual-level practices of boundary setting and self-control. Similarly, enhancing partner support at the interpersonal-level may fortify individual-level utilisation of biomedical tools, like PrEP or ART, to prevent HIV transmission during chemsex. Thus, given the interplay of practices across all three levels of action, interventions structured using this model can facilitate mutually reinforcing harm reduction strategies for individuals, interpersonal pairs, and communities, simultaneously.

Similarly, by targeting the moderating variables illustrated in this model, interventions can shape practices across all levels of action. For instance, interventions that increase agency in chemsex settings can help 1) individuals to set and keep boundaries, 2) interpersonal pairs to support each other with risk reduction strategies like PrEP and prevent from toxic co-dependent relationships that increase risk of drug dependency, and 3) communities to organise more supportive sessions where groups share responsibility for monitoring wellbeing during chemsex.

### Limitations

This review has several limitations to be considered alongside its findings. Firstly, most studies included in the review were conducted in high-income Western countries. Chemsex is defined and practiced differently in other areas of the world. Additionally, the cultural context of chemsex varies globally, as seen in Guadamuz and Boonmongkon’s study conducted in Thailand, where differential social status exists between participants and hosts, and influencing participants’ agency in chemsex settings (Guadamuz & Boonmongkon, 2018). Furthermore, aside from Lim et al.’s study of chemsex in Malaysia, all studies included in this review are from countries where homosexuality are legal (Lim et al., 2018). Harsh social and legal conditions of drug use and of homosexuality may expose GBMSM to different and/or additional risks, such as surveillance on apps, exploitation, and arrest, related to their participation in chemsex, and thus, GBMSM may adopt different practices to navigate these realities (Lasco & Yu, 2023a, 2023b; Tan et al., 2018). As such, our conceptual model for benefit and risk navigation must be tailored to incorporate these contextual experiences.

Secondly, our findings do not define and explore societal-level actions related to chemsex (the fourth level in the SEM). The studies included in this review explored GBMSM’s chemsex behaviours and did not discuss practices organised and actioned by GBMSM at the societal level to enhance chemsex benefits and reduce chemsex risks. However, several moderating factors, like social and institutional stigma and access to biomedical tools, may work at the societal level to influence lower-level actions of GBMSM and their ability to practice harm reduction. For example, in Southeast Asian settings, funding remits from international health aid bodies create structural barriers to clean injecting equipment, thus moderating men’s ability at the individual, interpersonal, and community levels to engage in this risk-reducing practice (Tan et al., 2018; Witzel et al., 2023).

Thirdly, this meta-ethnography does not include evidence from studies that assess chemsex practices in transactional sex settings or among communities other than GBMSM, such as transgender women. Individuals in these settings and from these communities may perceive different benefits and risks related to their engagement in chemsex, including financial compensation and coercion, gender-based violence, and gender and sexual affirmation, and thus may employ different strategies to navigate these risks and benefits (Brooks-Gordon & Ebbitt, 2021; Wang et al., 2023). Legal and social contexts that criminalise and stigmatise sex workers, transgender individuals, and other marginalised communities may shape individuals’ ability to pursue these strategies, further informing their practices.

## Conclusion

Historically, chemsex has been viewed as a disruption to and disinhibition from the conventions and stigmas of sex and sociality (Pienaar et al., 2020; Race et al., 2016). GBMSM who participate in chemsex, as described in this meta-ethnography, act at the individual, interpersonal, and community levels to reduce perceived risks of chemsex. These risks occur alongside benefits, and, in some cases, the practices used to navigate them are one in the same. Therefore, health promotion in the context of chemsex must look holistically at GBMSM’s varied perceptions and practices and avoid problematising chemsex through normative assumptions that chemsex is only associated with health harms (Pienaar et al., 2018). Recognizing the diverse pathways through which both benefits and harms can arise from chemsex practices may create a more favourable environment for harm reduction. In doing so, research on and health promotion for chemsex should centre the factors that moderate men’s practices—trust, agency, access, stigma, and setting—so that individuals, interpersonal pairs, and communities are empowered and equipped to enhance the benefits and the reduce risks of chemsex.

## Supplementary Material

Supplementary Material

## Figures and Tables

**Table 1:** Study characteristics.

Reference Number	Article	Country	Study Population	N	Data Collection Method(s)	Study Aim
1	Guadamuz & Boonmongkon, 2018	Thailand	GBMSM aged 18–29, able to converse in Thai, had used ice (methamphetamine) and had anal sex with another man in the last 6 months	40	40 repeated (3 times) narrative interviews 10 focus group discussions Systematic online observations on hook up websites and apps	“Use secrecy as an approach to better understand ice parties and provide implications for harm reduction efforts.”
2	Dennermalm et al., 2021	Germany and Sweden	GBMSM aged 18–46, Swedish citizen, currently or formerly a resident of Berlin or travel to Berlin at least twice per year, recreational drug use or sexual drug use during the past 3 years	15	15 one-on-one, semi-structured interviews	“Improve understanding of drug use, sex on drugs and harm reduction techniques among Swedish MSM who travel to Berlin in order to improve health among MSM using drugs”
3	Flores-Aranda et al., 2019	Canada	GBMSM aged 18+, reported methamphetamine use in the last 12 months or ceased methamphetamine use in for at least 12 months	17	2 focus groups, one (*n* = 9) with GBMSM currently using methamphetamines and one (*n* = 8) with GBMSM who had formerly used methamphetamines	“Document the perceptions of current and former methamphetamine users regarding their experiences of pleasure in relation to their use of this substance.”
4	Santoro et al., 2020	Spain	GBMSM aged 18+ year, residing in Madrid, having practiced chemsex at least once in the past 24 months	18	11 in-depth interviews 2 (*n* = 7) focus groups	“Show chemsex as a more complex set of practices than it is usually considered to be - to ‘open the black box’ of chemsex.”
5	Ahmed et al., 2016i	England	GBMSM aged 18+ years, living in Lambeth, Southwark, or Lewisham Boroughs, who had combined sex with another man/men and drugs (methamphetamine, GHB/GBL, and/or mephedrone) in the past 12 months	42	30 one-on-one, in-depth interviews 2 focus groups of six men each	“Describe the nature and operation of social norms relating to chemsex among gay men in South London, and identify public health implications”
6	Bourne et al., 2015ai	England	GBMSM aged 18+ years, living in Lambeth, Southwark, or Lewisham Boroughs, who had combined sex with another man/men and drugs (methamphetamine, GHB/GBL, and/or mephedrone) in the past 12 months	30	30 one-on-one, in-depth interviews	“Understand the experience of, or exposure to, harm relating to chemsex and the harm reduction services that might be required to address such issues”
7	Closson et al., 2018	United States	GBMSM aged 18+, HIV negative, who reported at least two episodes of condomless anal intercourse with another man while under the influence of methamphetamine, crack, powder cocaine, ecstasy, GHB, and/or poppers in the previous 3 months	40	40 one-on-one, semi-structured interviews	“Explore perceptions of PrEP adherence and preferences for PrEP dosing schedules among 40 HIV-negative MSM who reported recent concurrent recreational drug use and sexual risk.”
8	Drysdale et al., 2021ii	Australia	GBMSM aged 18+, reported methamphetamine use in the past 12 months, willing to discuss topics including sexual activity and illicit drug use	88	88 one-on-one, semi-structured interviews	“Examine how risk is understood and prioritised by gay and bisexual men who combine crystal use and sex, and we identify a range of risk reduction practices that they used, including some that may appear counter intuitive.”
9	Lim et al., 2018	Malaysia	GBMSM aged 18+, have engaged in oral or anal intercourse with another man in the last 6 months, have used recreational drugs (cocaine, methamphetamines, ecstasy, ketamine, erectile dysfunction drugs) in the last 3 months	20	20 one-on-one, in-depth interviews	“Understand the motivations for, and management of, methamphetamine use among MSM in Malaysia.”
10	Maxwell et al., 2022	United Kingdom (various countries)	GBMSM aged 18+, HIV-negative, residing in the UK, had engaged in chemsex in the previous 3 months, were currently using or had used PrEP in the previous 12 months	19	19 semi-structured, in-depth interviews	“Explore the biopsychosocial factors related to GBM’s chemsex experiences which influenced their motivation to use, access to and effective use of PrEP.”
11	Race et al., 2021	Australia	GBMSM aged 18+, reported injecting methamphetamine, willing to discuss topics including sexual activity and illicit drug use	13	13 in-depth, semi-structured interviews	“Explore how slamming has emerged as a sexual preference for some participants in chemsex scenes.”
12	Schroeder et al., 2022	Australia	GBMSM aged 18+ years, reporting any injection drug use (current or past)	19	19 in-depth interviews	“Generate an in-depth understanding of the social practice of injecting drug use among GBM in Australia.”
13	Souleymanov et al., 2019iii	Canada	GBMSM aged 18+ years, live in the Greater Toronto area, speak and read English, report use of drug (including methamphetamine, GHB, cocaine, ecstasy, ketamine, poppers) for sex in the last month	44	44 semi-structured interviews	“Explore how epistemic shifts associated with advancements in HIV biomedical sciences influenced these men’s perceptions of risk and their sexual and drug-related practices and provide a more nuanced understanding of how sexual and drug-related risk practices of these men are entangled with their search for pleasure.”
14	Souleymanov et al., 2021iii	Canada	GBMSM aged 18+ years, live in the Greater Toronto area, speak and read English, report use of drug (including methamphetamine, GHB, cocaine, ecstasy, ketamine, poppers) for sex in the last month	44	44 semi-structured interviews	“Examine the particular ways in which participants represented, constituted and made sense of various forms of social exclusion affecting themselves and others who PnP (party and play) and the resilience discourses of gay and bisexual men who PnP; that is, the particular ways in which participants spoke about and made sense of resilience, capitalised on this resilience to mitigate harms and resisted social exclusion.”
15	Bourne et al., 2015bi	England	GBMSM aged 18+ years, living in Lambeth, Southwark, or Lewisham Boroughs, who had combined sex with another man/men and drugs (methamphetamine, GHB/GBL, and/or mephedrone) in the past 12 months	30	30 one-on-one, in-depth interviews	“Explore HIV/STI transmission risk behaviour during the intentional combining of sex with mephedrone, GHB/GBL and methamphetamine”
16	Drysdale et al., 2020ii	Australia	GBMSM aged 18+, reported methamphetamine use in the past 12 months, willing to discuss topics including sexual activity and illicit drug	88	88 one-on-one, semi-structured interviews	“Destabilise the term chemsex so that a greater diversity and contingency of practice is captured.”
17	Herrijgers et al., 2020	Belgium	GBMSM aged 18+, able to express onself in Dutch or English, having intentionally used drugs (methamphetamine, mephedrone, GHB/GBL, XTC/MDMA, cocaine, NPS, and/or ketamine) to have sex within the past 12 months	20	20 one-on-one, semi-structured interviews	“Describe the experiences and needs of the local GBMSM community engaging in chemsex.”
18	Nimbi et al., 2020iv	Italy	GBMSM aged 18+, fluent Italian speaker, living in Italy, reporting at least one chemsex experience in their life	30	30 semi-structured interviews	“Investigate the experience lived by MSM who engage in chemsex in Italy, focusing on specific contexts, on the patterns of substance use, and on the perceived need for HR services.”
19	van Hout et al., 2019	Ireland	GBMSM aged 18+ years, attending session for support concerning their participation in the chemsex scene	10	10 semi-structured interviews	“Explore sexualised drug use pathways among gay, bisexual and other men who have sex with men experiencing physical and emotional health problems as consequence of their engagement with the sexualised drug use culture in Dublin, and who were seeking service supports to exit the Chemsex scene.”
20	Nimbi et al., 2022iv	Italy	GBMSM aged 18+, fluent Italian speaker, living in Italy, reporting at least one chemsex experience in their life	30	30 semi-structured interviews	“Have a better understanding of the sexual experience of MSM practicing chemsex under a psycho-sexological biopsychosocial perspective.”
21	Amaro, 2016	France	GBMSM aged 23 –30 years, living in Lyon or Paris, who use drugs (including injecting drugs) in combination with sex	25	25 one-on-one, in-depth interviews	“Provide a better understanding, based on ethnographic research and in-depth interviews, of how practices of injection drug use are entangled with the complex affective trajectories of young gay men.”
22	Leyva-Moral et al., 2023	Spain	GBMSM aged 18+ using chemsex support services at StopSida	30	12 one-on-one interviews 3 focus groups (*n* = 5, *n* = 5, and *n* = 8)	“Describe the practice of chemsex from the perspective of users to deepen the understanding of the factors associated with the practice, the perception of the impact on health, and prevention requirements.”
23	Milhet et al., 2019	France	GBMSM aged 18+ who report current engagement in chemsex	33	33 one-on-one in-depth interviews	“Explore chemsex from a ‘pleasurable’ frame of reference among gay men and other MSM in France.”

^i^ Articles originate from the same research study conducted by Bourne et al. in England. ^ii^ Articles originate from the same research study conducted by Drysdale et al. in Australia. ^iii^ Articles originate from the same research study conducted by Souleymanov et al. in Canada. ^iv^ Articles originate from the same research study conducted by Nimbi et al. in Italy.

**Table 2:** Summary of constructs.

Third-Order Labels	Third-Order Constructs	Second-Order Labels	Summary of Translation of First- and Second-Order Constructs	Contributing References
Individual control	Men try to control their exposure to risk through specific individual-level practices that draw boundaries around their chemsex experience. These practices can be more difficult to carry out if men lack agency in chemsex settings and access to specific health resources. Creating these boundaries and tailoring their practices allows men to have the type of experiences that they want, which enhances benefits of chemsex.	Preparing personal chemsex experience	Prepare ahead of time for physical recovery by buying food, electrolytes Planning things for after chemsex and creating exit strategies helps create personal boundaries Prepare doses ahead of time to avoid overdose Act of “getting everything in place” is erotic in its anticipation for people who inject	2, 4, 9, 10, 11, 12, 16, 17, 20, 22, 23
		Setting and keeping personal boundaries	Compartmentalise chemsex to avoid dependency Limit frequency of participation in chemsex Not learning to inject, not buying drugs, and not having drug paraphernalia creates boundary in life Not injecting, not sharing needles, not sharing toys, staying somewhat lucid help men to enjoy chemsex without anxiety of risks Difficult to exert boundaries when power imbalance between participants	1, 2, 3, 5, 6, 10, 11, 12, 13, 16, 17
		Utilising biomedical tools	CAI enhances pleasure More confident to engage in CAI by taking PrEP People who are HIV-positive have responsibility to take HIV medication and people who are HIV-negative have responsibility to take PrEP Testing regularly, DoxyPEP, and using clean injecting equipment reduce risk of HIVSTIs/BBVs Not all men have access to PrEP or other biomedical harm reduction resources	2, 3, 7, 10, 13, 15, 16, 17, 20, 21
		Structuring use of drugs	Men control dosing to maximise impact Dosing correctly is important to avoid overdose (especially from GHB/GBL) Keep dosing logs/schedules Take different drugs for different desired effects Methamphetamine can help reverse GHB overdose Erectile dysfunction medication and poppers help fulfil sexual fantasies Slamming can provide more intense pleasure and confine chemsex experience Lack of agency over which type of drugs/route of administration they use when drugs are supplied/parties organised by hosts	2, 3, 5, 11, 13, 18, 19, 20, 23
Interpersonal relationships	Practices are negotiated and supported between pairs of men through different relationships to keep each other safe and satisfied. When individuals lack agency and trust in these relationships, practices may actually enhance risks.	Leaning on partners	Watch out for one another Respect personal boundaries/set boundaries for each other Make each other feel safe Can enter into symbiotic relationships that further each other’s dependence on chemsex	1, 8, 16, 21
		Injecting/being injected by others	Enhances emotional intimacy between partners Can show compassion and care when injecting others Injection by someone who is more experienced to avoid potential risks of faulty injection Learn to inject themselves when they do not trust others to do it correctly	11, 12, 16, 17
Community care	In group chemsex settings, men care for one another to ensure their safety. This is dependent on trust, agency, and stigma felt in these group settings. Feeling safe and cared for by others also enhances men’s ability to experience pleasure and community connectedness in chemsex settings.	Group organising	Hosts organise chemsex parties by HIV status, type of drug, route of administration, serosorting Discussion of sexual and personal boundaries prior to drug use Group logs for dosing schedules and reminders for things like medication, food, dosing	2, 4, 5, 7, 8, 9, 10, 12, 16, 18, 19
		Monitoring others’ safety	Group organising can make people feel exclusive, part of community Practicing chemsex together develops trust and attentiveness to others’ needs Trusted groups of friends support PrEP and HIV medication adherence Hosts have a responsibility to watch out for others’ safety in anonymous sessions Caretaker role in groups can be its own sexual fantasy and erotic Keeping networks small ensures trust Makes people feel safe and creates sense of belonging	1, 2, 6, 13, 14, 16, 17, 18, 19, 20
		Teaching and learning	Helping others with dosing strategies and drug effects Sharing “home remedies” for overdose and bad experiences Teaching others to inject can be erotic and empowering Not teaching others to inject can protect them from risk	1, 2, 6, 11, 14, 18, 22
