# Supplementary material for: Exploring practices to enhance benefits and reduce risks of chemsex among gay, bisexual, and other men who have sex with men: A meta-ethnography
Source: Int J Drug Policy. Author manuscript; Available in PMC 2025 Nov 22. (PMC7618392; doi:10.1016/j.drugpo.2024.104398)
Supplement: Supplementary Material [file EMS210917-supplement-Supplementary_Material.docx]

**Supplementary Material A. MEDLINE Search Strategy**

| **Concept 1:** Sample Population [GBMSM] | Keywords | GBMSM* OR MSM OR "men who have sex with men" OR "men having sex with men" OR gay* OR bisexu#l men OR homosexual* OR bicurious* or (minorit* adj3 sexual*) OR bisexual* OR queer OR non-heterosexual OR non-heteronormative OR transmen OR trans men OR transmale* OR transgender men OR transgender male* OR GLBT OR GLB OR GLBTQ* OR LGB OR LGBT LGBTQ* |
| --- | --- | --- |
|  | Subject Headings | Homosexuality/ OR exp Bisexuality/ OR Homosexuality, Male/ OR exp Sexual and Gender Minorities/ |
| **Concept 2:** Phenomenon of Interest  [Sexual Intercourse] | Keywords | chemsex* OR slam* OR slamsex* OR "party and play" OR PNP OR "party 'n play" OR "high and horny" OR HNH OR "high 'n horny" OR SDU OR (sex* adj3 drug*) OR sexual* substance* OR sexual intercourse OR sexual encounter* OR sexual context* OR sexual setting* OR sexual activiti* OR sexual practice* OR sexual behavio* OR anal sex OR anal intercourse OR group sex OR sex party OR sexual partner* OR sex partner* OR casual partner* OR regular partner* OR multiple partners |
|  | Subject Headings | Sexual Behaviour/ OR exp Coitus/ OR HIV Serosorting/ OR Orgasm/ OR Masturbation/ OR Sex Work/ OR Safe Sex/ OR Sexual Harassment/ OR Sexuality/ OR Unsafe Sex/ OR Sexual Partners/ |
| **Concept 3:** Phenomenon of Interest  [Substance Use] | Keywords | stimulant* OR amphetamine* OR methamphetamine* OR "tina" OR "crystal" OR "meth" OR "gamma-Hydroxybutyric acid" OR "GHB" OR "GBL" OR "GHB/GBL" OR "Gina" OR "Liquid E" OR cathinone* OR mephedrone OR "M-Cat" OR "Meow-Meow" OR ecstasy OR MDMA OR "molly" OR "mandy" OR cocaine* OR ketamine* OR 5-methoxy-N OR "foxy-5" OR "Tramadol" OR opiate* OR methaqualone OR quaalude* OR barbiturate* OR "new psychoactive substance" OR "new psychoactive substances" OR "NPS" OR psychotropic drug* or psychotropic substance* OR hallucinogen* OR recreational drug* OR club drug* OR party drug* OR street drug* OR illicit drug* OR illegal drug* OR synthetic drugs* OR synthesi#ed drug* OR designer drug* OR customi#ed drug* OR recreational substance* OR illicit substance* OR illegal substance* OR synthetic substance* OR synthesi#ed substance* OR customi#ed substance* OR designer substance* OR drug-related OR drug abus* OR drug addict* OR drug dependenc* OR (drug* adj4 us*) OR substance-related OR substance abus* OR substance addict* OR substance dependenc* OR (substance* adj4 us*) |
|  | Subject Headings | exp Amphetamines/ OR exp Methamphetamine/ OR Substance-Related Disorders/ OR Amphetamine-Related Disorders/ OR Cocaine-Related Disorders/ OR Drug Overdose/ OR Psychoses, Substance-Induced/ OR Substance Abuse, Intravenous/ OR Substance Abuse, Oral/ OR Substance Withdrawal Syndrome/ OR exp Illicit Drugs/ OR exp Synthetic Drugs/ OR Psychotropic Drugs/ OR exp Hallucinogens/ OR exp Tranquilizing Agents/ OR exp Cocaine/ OR Cocaine Smoking/ OR Ketamine/ OR Self Medication/ |
| **Concept 4:** Design/Methods  [Qualitative Research] | Keywords | "semi-structured" or semistructured or unstructured or informal or "in-depth" or indepth or "face-to-face" or structured or (guide adj3 interview* or discussion* or questionnaire*) or focus group* or qualitative or "key informant" |
|  | Subject Headings | exp Qualitative Research/ OR exp Interview/ OR exp Focus Group/ |
